# Supplementary material for: App-based COVID-19 syndromic surveillance and prediction of hospital admissions in COVID Symptom Study Sweden
Source: Nat Commun. 2022 Apr 21;13:2110. doi: 10.1038/s41467-022-29608-7 (PMC9023535; doi:10.1038/s41467-022-29608-7)
Supplement: Supplementary file 2 — Reporting Summary [file 41467_2022_29608_MOESM2_ESM.pdf]

Corresponding author(s): Tove Fall

Last updated by author(s): Mar 17, 2022

Reporting Summary

Nature Portfolio wishes to improve the reproducibility of the work that we publish. This form provides structure for consistency and transparency in reporting. For further information on Nature Portfolio policies, see our Editorial Policies and the Editorial Policy Checklist.

Statistics

For all statistical analyses, confirm that the following items are present in the figure legend, table legend, main text, or Methods section.

n/a	Confirmed	
☐	☒	The exact sample size (n) for each experimental group/condition, given as a discrete number and unit of measurement
☐	☒	A statement on whether measurements were taken from distinct samples or whether the same sample was measured repeatedly
☒	☐	The statistical test(s) used AND whether they are one- or two-sided<br>*Only common tests should be described solely by name; describe more complex techniques in the Methods section.*
☐	☒	A description of all covariates tested
☒	☐	A description of any assumptions or corrections, such as tests of normality and adjustment for multiple comparisons
☐	☒	A full description of the statistical parameters including central tendency (e.g. means) or other basic estimates (e.g. regression coefficient) AND variation (e.g. standard deviation) or associated estimates of uncertainty (e.g. confidence intervals)
☒	☐	For null hypothesis testing, the test statistic (e.g. F, t, r) with confidence intervals, effect sizes, degrees of freedom and P value noted<br>*Give P values as exact values whenever suitable.*
☒	☐	For Bayesian analysis, information on the choice of priors and Markov chain Monte Carlo settings
☐	☒	For hierarchical and complex designs, identification of the appropriate level for tests and full reporting of outcomes
☐	☒	Estimates of effect sizes (e.g. Cohen's d, Pearson's r), indicating how they were calculated

Our web collection on statistics for biologists contains articles on many of the points above.

Software and code

Policy information about availability of computer code

Data collection	Data for Sweden and UK presented in the study was collected by ZOE Limited using a smartphone app. Source code for this app is not publicly available.
Data analysis	All code necessary for the replication of our results, including reproducibility instructions, is available on GitHub at https://github.com/ulfha881/App-based-COVID-19-syndromic-surveillance-and-prediction-of-hospital-admissions-The-COVID-Symptom-S, or, in archived form, at https://doi.org/10.5281/zenodo.6069218. R and Stata codes are provided that will allow readers to generate Figures 2–5 from Source Data. Codes to calculate prevalence estimates and the prediction models are provided, but without unmodified individual level datasets, they can't be executed in full.<br>Demographic data from 2021 was available from Statistics Sweden. The 95% confidence intervals (95% CI) for predictions were generated using the function ageadjust.direct from the epitools package in R (version 3.6.1).

For manuscripts utilizing custom algorithms or software that are central to the research but not yet described in published literature, software must be made available to editors and reviewers. We strongly encourage code deposition in a community repository (e.g. GitHub). See the Nature Portfolio guidelines for submitting code & software for further information.

Data

Policy information about availability of data

All manuscripts must include a data availability statement. This statement should provide the following information, where applicable:

- Accession codes, unique identifiers, or web links for publicly available datasets
- A description of any restrictions on data availability
- For clinical datasets or third party data, please ensure that the statement adheres to our policy

Primary data in this study were collected by ZOE Limited and provided to CSSS under a data-sharing agreement. Additional anonymized data originated from the National Board of Health and Welfare, the Public Health Agency of Sweden, Statistics Sweden, and NOVUS. Restrictions apply to the availability of these data, which were used under license and ethical approval and are not publicly available. Pseudonymized individual-level data are, however, available from the authors upon reasonable request and with written permission from the Swedish Ethical Review Authority. These data may only be used for research, and are not available for commercial use. Data requests will be processed within one month if a written ethical approval is submitted to the corresponding author. Group-level prevalence estimates calculated from individual-level data, hospitalization data, and cases of COVID positivity are available as the Source Data (Figures.zip), which were used to generate Figures 2–5. We provide mock CSSS individual level datasets, that can be used together with the code. These data do not represent real observations. The Source Data that support the findings of this study are available on GitHub at https://github.com/ulfha881/App-based-COVID-19-syndromic-surveillance-and-prediction-of-hospital-admissions-The-COVID-Symptom-S, or, in archived form, at https://doi.org/10.5281/zenodo.6069218.

Field-specific reporting

Please select the one below that is the best fit for your research. If you are not sure, read the appropriate sections before making your selection.

[x] Life sciences [ ] Behavioural & social sciences [ ] Ecological, evolutionary & environmental sciences

For a reference copy of the document with all sections, see nature.com/documents/nr-reporting-summary-flat.pdf

Life sciences study design

All studies must disclose on these points even when the disclosure is negative.

Sample size	COVID Symptom Study Sweden (CSSS) was launched in Sweden on April 29, 2020 to provide COVID-19 syndromic surveillance data and to build a large-scale repeated measures database for COVID-19 research. All individuals ≥18 years living in Sweden with access to a smart device were eligible to participate in the CSSS after downloading the app and providing informed consent. No sample size calculation was performed before the launch, but the app had been launched in the UK March 24, 2020, and 1.5 million UK participants had been recruited within the five days after launch. Here, we included data from April 29, 2020 to February 10, 2021 and the final Swedish CSSS study population consisted of 143,531 individuals.<br>Using the CSSS data together with Swedish national health register data, we developed a model to predict subsequent regional number of COVID-19 hospital admissions. This prediction model was validated in an English dataset that consisted of data from ZOE COVID Study (previously known as COVID Symptom Study UK) and English health register data. We included data from ZOE COVID Study from March 30, 2020 to January 31, 2021, and the final study English population consisted of 1,888,416 individuals.
Data exclusions	Participants were excluded from the CSSS analyses if they: 1) had never submitted a daily report (n=5,931), 2) had missing age or reported an age <18 or >99 years (n=801), or 3) stated their sex as other/intersex (n=236) as this sample size was insufficient for a separate analysis. Participants whose last report was within seven days of joining the study (n=45,483) or did not provide a valid postal code (n=7,310) were excluded from the prevalence estimation, but included in model training if they had reported a PCR test and had submitted at least one symptomatic daily report within seven days preceding or on the test date (n=967). These same exclusion critera were applied to the ZOE COVID Study dataset.
Replication	There are five main steps to the analyses reported in the study, and we developed two separate models which were both replicated:<br>Step 1) We developed a model to estimate the individual probability of a positive COVID-19 PCR test, utilizing information from symptomatic CSSS study participants who had reported at least one PCR test result. This prediction model was validated in an external Swedish dataset from the separate CRUSH Covid study (n=943; October 18, 2020 to February 10, 2021).<br>Step 2) We employed the model from Step 1 to assess daily individual probability of symptomatic COVID-19 in all CSSS study participants.<br>Step 3) We used a weighted average of individual probabilities from Step 2 to estimate daily regional COVID-19 prevalence in the general population in Sweden.<br>Step 4) We developed an iterative time-updated prediction model to assess if the regional prevalence estimates from Step 3 could be used together with current hospital data for prediction of regional COVID-19 hospital admissions seven days ahead.<br>Step 5) We sought to validate the CSSS-based hospitalization prediction model in England by repeating Steps 2 and 3 and parts of Step 4 in an English dataset that consisted of information from ZOE COVID Study and regional English health register data.
Randomization	COVID Symptom Study Sweden is an observational study, and no randomization has been performed.
Blinding	Blinding was not appicable in our study, as it is not an intervention study. Our study is an observational symptomatic surveillance study based on voluntary individual participation.

Reporting for specific materials, systems and methods

We require information from authors about some types of materials, experimental systems and methods used in many studies. Here, indicate whether each material, system or method listed is relevant to your study. If you are not sure if a list item applies to your research, read the appropriate section before selecting a response.

Materials & experimental systems

n/a	Involved in the study
☒	☐ Antibodies
☒	☐ Eukaryotic cell lines
☒	☐ Palaeontology and archaeology
☒	☐ Animals and other organisms
☐	☒ Human research participants
☒	☐ Clinical data
☒	☐ Dual use research of concern

Methods

n/a	Involved in the study
☒	☐ ChIP-seq
☒	☐ Flow cytometry
☒	☐ MRI-based neuroimaging

Human research participants

Policy information about studies involving human research participants

Population characteristics	In this study, we included data from COVID Symptom Study Sweden from April 29, 2020 to February 10, 2021. Participant attributes are found in Supplementary Table 1. The final study population consisted of 143,531 individuals. The median duration of study participation was 151 days (inter-quartile range [IQR] 52–252), with a median of 43 days with submitted reports (IQR 13–119). Of all participants, 30% reported at least one COVID-19 PCR test, and 6% of women and 4% of men reported at least one positive test result. The cohort included a larger proportion of women, fewer people ≥65 years, and fewer smokers than the general population, while the prevalence of obesity was similar. Participants further resided in postal code areas with less deprivation, similar proportions of inhabitants with foreign background, and higher population densities, as compared to the general population.<br>Using the CSSS data together with Swedish national health register data, we developed a model to predict subsequent regional number of COVID-19 hospital admissions. This prediction model was validated in an English dataset that consisted of data from ZOE COVID Study (previously known as COVID Symptom Study UK) and English health register data. We included data from ZOE COVID Study from March 30, 2020 to January 31, 2021, and the final study English population consisted of 1,888,416 individuals. The median duration of study participation in ZOE COVID Study was 182 days (inter-quartile range [IQR] 62-305), with a median of 43 days with submitted reports (IQR 13–119). Similar to the Swedish setting, the English cohort included a larger proportion of women and fewer people ≥65 years compared to the general population.
Recruitment	All individuals aged ≥18 living in Sweden with access to a smart device have been eligible to participate in the CSSS by downloading the app and providing informed consent. Information about the study has been publicably available via Lund University and Uppsala University (home pages, news letters, social media) outlets, and the study has also been mentioned in national press. ZOE COVID Study has similarly invited all individuals aged ≥18 living in the UK to participate with information about the study publicably available through websites and social media. ZOE COVID Study has further been publicably endorsed by NHS Wales and NHS Scotland.
Ethics oversight	The Swedish Ethical Review Authority has approved COVID Symptom Study Sweden (DNR 2020-01803 with addendums 2020-04006, 2020-04145, 2020-04451, 2020-07080, and 2021-02316) and CRUSH Covid (DNR 2020-07080, and DNR 2020-04210 with addendum 2020-06315). In the United Kingdom, the ZOE COVID Study has been approved by King's College London (KCL) ethics committee REMAS ID 18210, review reference LRS-19/20-18210. All participants in the COVID Symptom Study Sweden and in ZOE COVID Study in the UK have provided informed consent. Participants have not been compensated for their participation.

Note that full information on the approval of the study protocol must also be provided in the manuscript.
